# Construction of the structural equation model of stigma, self-disclosure, social support, and quality of life of breast cancer patients after surgery—a multicenter study

**DOI:** 10.3389/fonc.2023.1142728

**Published:** 2023-05-18

**Authors:** Liuxiu Bu, Xisui Chen, Shaoyan Zheng, Guanhua Fan

**Affiliations:** ^1^ Shantou University Medical College, Shantou, China; ^2^ Department of Nursing, First Affiliated Hospital of Shantou University Medical College, Shantou, China

**Keywords:** breast cancer, stigma, self-disclosure, social support, quality of life, structural equation model

## Abstract

**Purpose:**

Stigma is common in patients with breast cancer after surgery, which has a negative impact on the quality of life (QOL). This study aimed to investigate the QOL of breast cancer patients after surgery and to analyze the multiple chains mediating effects of self-disclosure and social support between stigma and QOL.

**Methods:**

A total 292 patients of breast cancer patients after operation were recruited in this study. A questionnaire survey was conducted using the general information questionnaire, the consumer experiences of stigma questionnaire (CESQ), the distress disclosure index(DDI), the perceived social support scale(PSSS), and the functional assessment of cancer therapy-breast(FACT-B). Path analysis was conducted to test the hypothesized serial multiple mediation model.

**Results:**

The total scores of stigma, self-disclosure, social support and QOL were 15 (10 ~ 22), 39 (31 ~ 46), 58 (50 ~ 67) and 88 (74 ~ 104) respectively. QOL of breast cancer patients after the operation was negatively correlated with stigma (p < 0.01), and positively correlated with self-disclosure and social support (p < 0.01). Self-disclosure and social support played a complete mediating effect between stigma and QOL, and the total mediating effect value was 85. 87%.

**Conclusions:**

Self-disclosure and social support play a complete intermediary role between stigma and QOL. In order to improve the quality of life of patients, medical staff should pay attention to the assessment of stigma, encourage patients to express their emotions, and encourage their families and friends to respond to their expression and needs of patients.

## 1 Backgrounds

According to the latest Cancer Statistics Report in 2021 (1), breast cancer accounted for the first morbidity of women’s cancer, which posed a serious threat to women’s health and life. Surgery remained the mainstay of treatment for breast cancer patients (2), but increasingly innovative screening techniques allowed early detection of the disease and with the development of better treatment options, the 5-year survival rate of breast cancer has been improved (3). In China, breast-conserving surgery and adjuvant radiotherapy for patients with early-stage breast cancer can make the 5-year overall survival rate of patients > 80% (4). The survival time of breast cancer patients had been prolonged. However, the interpersonal relationship, body image, and psychological status of breast cancer patients after surgery had been affected, which were closely related to the QOL (5, 6). Research had revealed that QOL had become an important outcome measure in breast cancer clinical research and survival research, and could be used as a predictor of mortality rate in breast cancer survivors (7). Research had shown that patients with breast cancer after surgery generally experienced stigma, which had an adverse impact on the QOL of patients (8–10), and patients’ self-disclosure and social support could improve the QOL, which had a positive impact (11–13). The stigma of breast cancer patients is negatively correlated with self-disclosure and social support (14). Self-disclosure could enhance the benefits of social support and promoted their mental health (15). Based on the above analysis of the logical relationship between breast cancer patients’ stigma, social support and self-disclosure variables, the purpose of this study is to investigate the relationship among stigma, self-disclosure, social support, and QOL in breast cancer patients after surgery, and to construct the chain mediation model of self-disclosure and social support. Exploring the influencing factors of QOL of patients, and providing suggestions for intervention programs to improve the QOL for breast cancer patients after surgery.

## 2 Methods

### 2.1 Participants and procedures

**Figure 1:**
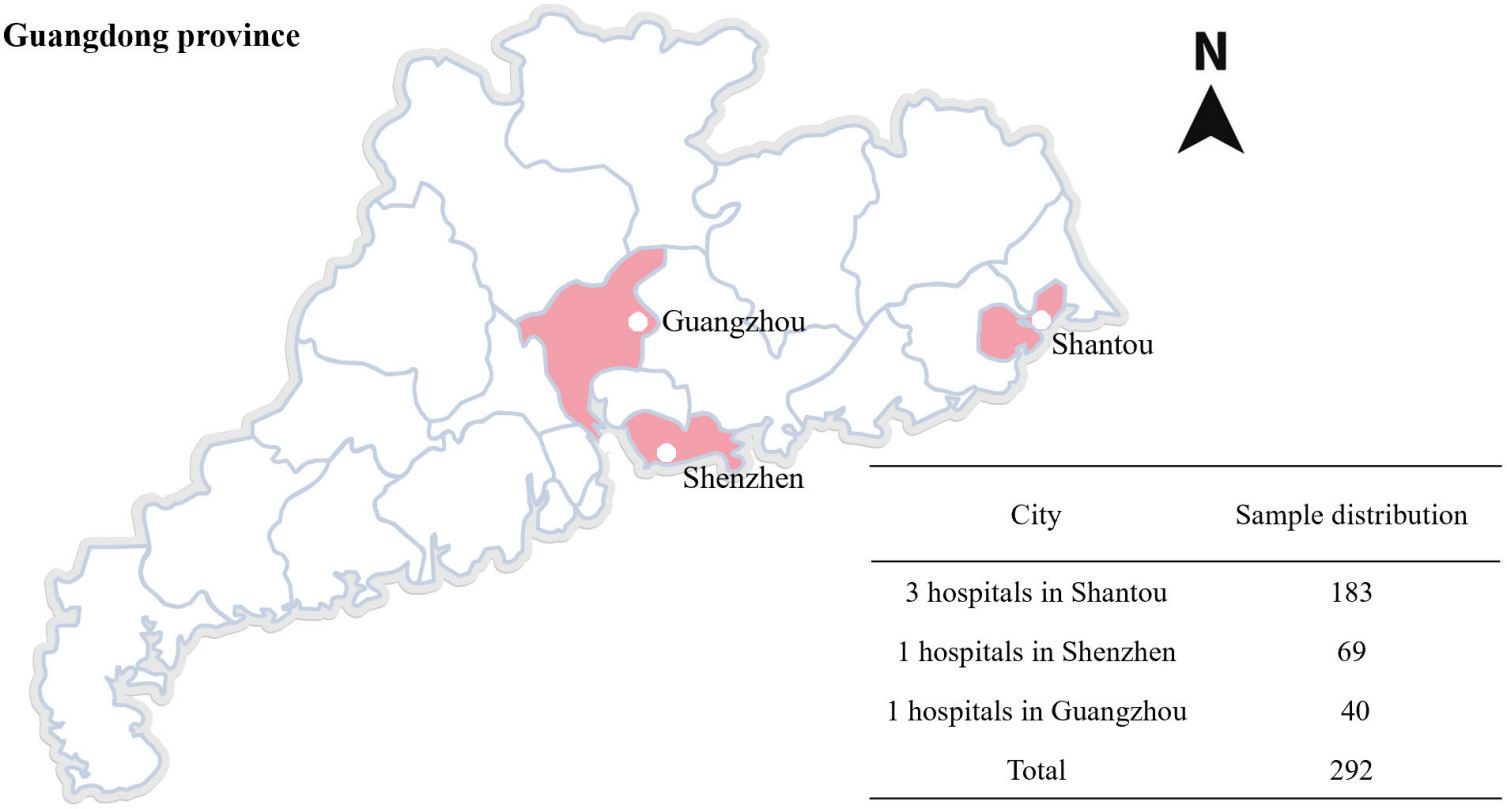
Data collection distribution map.

A total of 292 breast cancer patients from 5 hospitals in Guangdong Province (3 hospitals in Shantou, 1 hospital in Guangzhou, and 1 hospital in Shenzhen) from March 2021 to March 2022 were selected. To increase the sample size and combine with clinical practice, a convenient sampling method is adopted, and the number of participants in each hospital was evenly distributed as far as possible (**Figure 1**).

Inclusion criteria: (1) pathological diagnosis of breast cancer (stage 0, I, II, III, IV); (2) patients accepted mastectomy, breast-conserving surgery, or breast reconstruction surgery;(3)age ≥ 18 years old; (4) voluntarily participate in this study on the premise of informed consent; (5)clear awareness, reading comprehension and expression ability; (6)awareness of their condition. Exclusion criteria: (1)combined with other malignant tumors or recurrence of breast cancer; (2)mental disorders, unable to cooperate; (3)critical condition, unable to understand or answer questions clearly; (4)combined with myocardial infarction, heart failure and other serious diseases affecting the QOL. Sample size calculation: Combined with popular rules-of-thumb and Monte Carlo analysis method, the minimum sample size is 265 (16–18).

### 2.2 Measures

#### 2.2.1 Sociodemographic and medical variables

Basic information such as gender, age, marital status, educational level, and religious belief was self-reported. Disease staging, pathological classification, and surgical methods were extracted from medical records.

#### 2.2.2 Consumer experiences of stigma questionnaire

The questionnaire was developed by Wahl et al. in 2013 to provide a tool for assessing stigma in breast cancer patients. This study adopted the Chinese version of the CESQ (19), which mainly included two aspects: the stigma of interpersonal communication and experience of discrimination, with a total of 9 items. Grade 0-5 scoring method was adopted: 0 points (never) -5 points (often), 0-45 points. The higher the score, the higher the level of stigma. In this study, the total Cronbach’s coefficient of CESQ was 0.942.

#### 2.2.3 Distress disclosure index

DDI was used (20), which consisted of 12 items and was scored by the Likert 5-level scoring method, with a total score of 12-60 points. The higher the DDI score, the higher the self-disclosure level. 12-29 points were low self-disclosure, 30-44 points were medium self-disclosure, and 45-60 points were high self-disclosure. In this study, the total Cronbach’s coefficient of DDI was 0.942.

#### 2.2.4 Perceived social support scale

PSSS was a 12-item scale developed by Blumenthal (21) in 1987. This scale was composed of three subscales: family support, friend support, and other support, and each subscale contained four items. Using the Likert 7-level scoring method, 1-7 points represented “extremely disagree” to “extremely agree”. The higher the score, the higher the level of social support, 12-36 points for low support level, 37-60 points for medium support level, and 61-84 points for high support level. In this study, the total Cronbach’s Coefficient of PSSs was 0.903, and the Cronbach’s coefficients of each subscale were 0.926, 0.945, and 0.903 respectively.

#### 2.2.5 Functional assessment of cancer therapy-breast

The 36-item of Functional Assessment of Cancer Therapy-Breast scale (22) was used to measure participants’ QOL, including physiological status, social/family status, and emotional status, and functional status and additional concerns about breast cancer five dimensions. All items were rated using a 5-point Likert scale, and the full score was 144 points. The higher the score, the higher the QOL of patients. In this study, the total Cronbach’s coefficient was 0.921, and the Cronbach’s coefficient of each dimension ranged from 0.455 to 0.914.

### 2.3 Data collection and analysis

This study was a cross-sectional design and had been approved by the ethics committee of the hospital. Before the study, researchers were trained and assessed in a unified way, and the researchers were required to use unified guidelines in the survey process. The subjects formally conducted a questionnaire survey after oral or signed informed consent. The completed questionnaire was checked and taken back by the researchers on the spot. Questionnaire exclusion criteria: questionnaire blank ≥ 15%; the answer was single and the content was contradictory. A total of 300 questionnaires were distributed, excluding 8 invalid questionnaires, 292 valid questionnaires were recovered, and the effective recovery rate was 97.3%. Descriptive statistics and correlations were performed in SPSS 26.0. The descriptive data were presented as mean ± SD for variables obeying normal distribution, Md (P25, P75) for variables not obeying normal distribution. The enumeration data were expressed by frequency and constituent ratio. In this study, the scores of stigma, social support, self-disclosure, and quality of life do not obey normal distribution, however, the scores of demographic characteristics are expressed by Md (P25, P75), and the correlation analysis is conducted by Spearman rank correlation analysis. Amos 26. 0 was used to construct the structural equation model, the bootstrap was used to evaluate the direct and indirect effects, and the effects of each path were tested. All tests were performed two-sided, and a p-value of less than 0.05 was considered a significant level.

## 3 Results

### 3.1 General information of patients

**Table 1** showed that the age of 292 patients with breast cancer who participated in the study was mainly 46-69 years old (65.1%), the education of the participants was generally junior college or below (72.9%), and only 26 patients had bachelor’s degree or above (8.9%); Among the religious beliefs, 68.2% of the patients had no religious beliefs, and 11.3% believed in Buddhism; Among the marital status, 91.4% were married; In the economic situation, 46.6% of patients were in the balance of payments. Among 292 cases of breast cancer, 282 cases (96. 6%) were invasive cancer, mastectomy was performed in 183 patients (62. 7%), and breast-conserving surgery was performed in 97 patients (33. 2%).

**Table 1:** Demographic and clinical characteristics of participants (n=292).

Characteristics	Category	N	Percentage (%)
Age (years)	<18	0	0
	18~45	93	31.8
	46~69	190	65.1
	>69	9	3.1
Education	Illiterate	53	18.2
	Below college	213	72.9
	College degree	24	8.2
	Post-graduate degree	2	0.7
Religion	No religion	199	68.1
	Buddhism	33	11.3
	Christian	6	2.1
	Taoism	1	0.3
	Tudi Gong	53	18.2
Marital	Unmarried	6	2.1
	Married	267	91.4
	Divorce	9	3.1
	Widowed	10	3.4
Economics	Slight surplus	83	28.4
	break even	136	46.6
	break the pale	73	25.0
Medical insurance	Yes	267	91.4
	No	25	8.6
Family history	Yes	12	4.1
	No	280	95.9
Time since surgery (months)	<1	88	30.1
	1~2	61	20.9
	>2	143	49.0
Stage of breast cancer	Stage I	59	20.2
	Stage II	140	47.9
	Stage III	66	22.6
	Stage IV	9	3.1
	Stage 0	18	6.2
Pathological classification	Invasive carcinoma	282	96.6
	Non invasive carcinoma	10	3.4
Surgery type	Mastectomy	183	62.7
	Breast conservation	97	33.2
	Breast reconstruction	12	4.1
Chemotherapy	Yes	206	70.5
	No	86	29.5
Targeted therapy	Yes	72	24.7
	No	220	75.3

### 3.2 The scores and correlation analysis of stigma, self-disclosure, social support, and QOL

The total score of CESQ was 15 (10~22), the total score of DDI was 39 (31~46), the total score of PSSS was 58 (50~67); The total score of FACT-B was 88 (74~104), and the 5 dimensions were: physiological status, 21 (19~23); social/family status, 17 (11~22); emotional status, 16 (13~19); functional status, 13 (8~17); additional concerns, 24 (21~26), respectively (**Table 2**). The correlation coefficients among the stigma, self-disclosure, social support, and QOL are shown in **Table 3**. The total scores of stigma, social support, self-disclosure, and QOL of patients were correlated with each other, and the correlation was statistically significant (P < 0.01). QOL was negatively correlated with stigma(r=-0.518, *p*<0.01), and positively correlated with self-disclosure(r=0.502, *p*<0.01) and social support(r=0.492, *p*<0.01). Stigma was negatively correlated with self-disclosure(r=-0.645, *p*<0.01) and social support(r=-0.564, *p*<0.01); Self-disclosure was positively correlated with social support(r=0.710, *p*<0.01).

**Table 2:** Scores for the consumer experiences of stigma questionnaire (CESQ), distress disclosure index (DDI), perceived social support scale (PSSS), functional assessment of cancer therapy-breast (FACT-B).

Scale	Items	Actual Range	M (*P*25˜*P*75)
**CESQ**	9	0-45	15 (10~22)
**DDI**	12	12-60	39 (31~46)
**PSSS**	12	0-84	58 (50~67)
**FACT-B**	36	0-144	88 (74~104)
Physiological status	7	0-28	21 (19~23)
Social/family status,	7	0-28	17 (11~22)
Emotional status,	6	0-24	16 (13~19)
Functional status	7	0-28	13 (8~17)
Additional concerns	9	0-36	24 (21~26)

**Table 3:** Bivariate correlations of study variable (*n*=292).

	1	2	3	4	5	6	7	8	9
1.stigma	1	-.645^**^	-.564^**^	-.518^**^	-.309^**^	-.456^**^	-.414^**^	-.458^**^	-.357^**^
2.Self- disclosure	–	1	.710^**^	.502^**^	.308^**^	.456^**^	.461^**^	.410^**^	.317^**^
3.Social support	–	–	1	.492^**^	.306^**^	.507^**^	.452^**^	.350^**^	.321^**^
4.Quality of life	–	–	–	1	.545^**^	.831^**^	.725^**^	.851^**^	.698^**^
5. Physiological	–	–	–	–	1	.220^**^	.451^**^	.323^**^	.423^**^
6. Social	–	–	–	–	–	1	.488^**^	.728^**^	.490^**^
7. Emotional	–	–	–	–	–	–	1	.464^**^	.480^**^
8. Functional	–	–	–	–	–	–	–	1	.439^**^
9. Additional	–	–	–	–	–	–	–	–	1

^**^:p<0.01.

### 3.3 Structural equation model of stigma, self-disclosure, social support, and QOL

**Figure 2:**
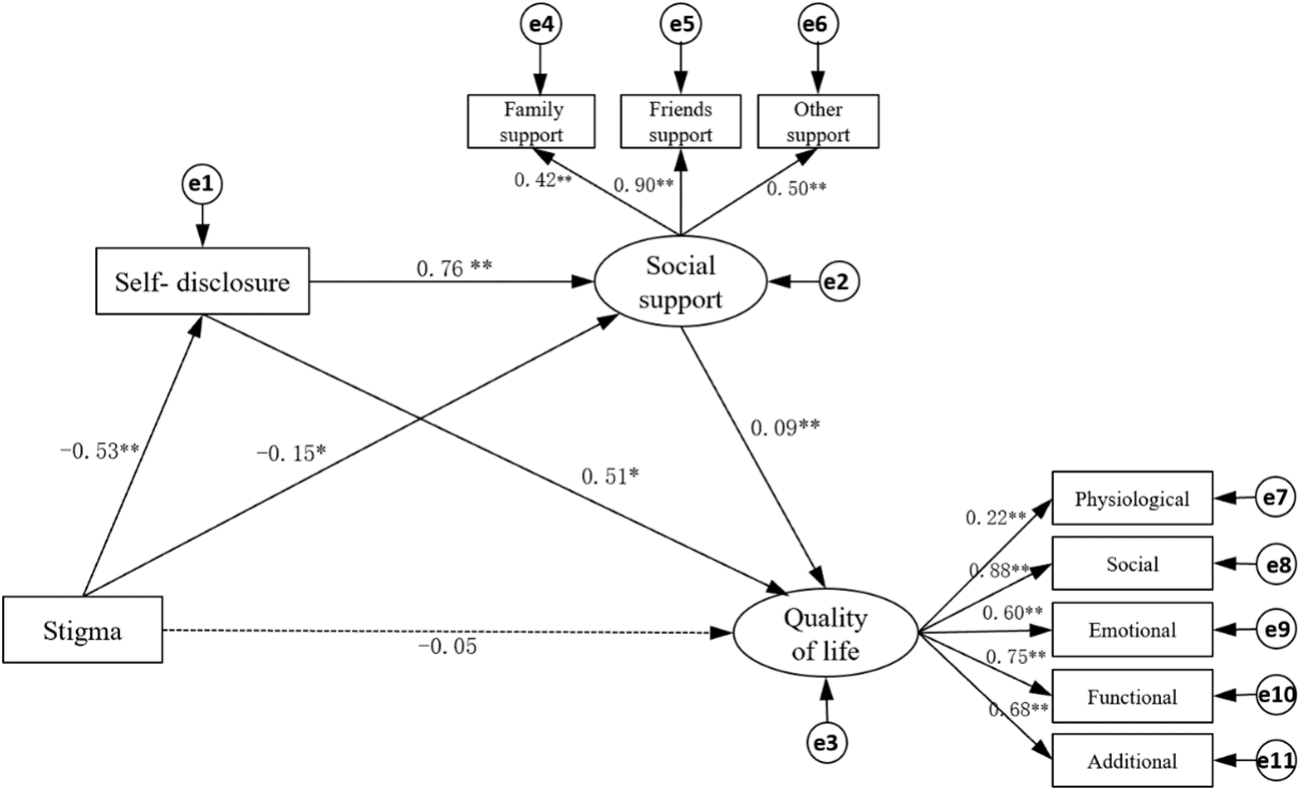
Structural equation model of quality of life of breast cancer patients. The dotted lines mean “not Significant”. The numbers represent correlation. *P < 0.05, ** p < 0.001.

Through a large number of literature research and correlation analysis results, it is hypothesized that QOL can be directly affected by stigma and indirectly affected by self-disclosure and social support. The maximum likelihood method was used to fit the model structure, and the model was corrected according to the correction index. The fitting results of the model: *X^2^/df=*2.512, goodness of fit(GFI)=0.963, adjusted goodness of fit (AGFI)=0.906, root mean square error of approximation(RMSEA)=0.072, normed fit index(NFI)=0.958, comparative fit index(CFI)=0.974, which indicated that the fitting degree of the model was good (6) (**Table 4**). **Figure 2** shows that the direct effect of stigma on QOL is not significant ( *β*=- 0.05, *p*>0.05), and stigma can indirectly affect the QOL through self-disclosure and social support. **Table 5** has shown the path coefficients among the variables detailedly. The results of multiple mediating effect analyses showed that self-disclosure and social support played a complete mediating role in the stigma and QOL of breast cancer patients after surgery. The mediating effect value was -0.322, accounting for 85.87% of the total effect, of which the mediating effect of self-disclosure accounted for 72.27%, the mediating effect of social support accounted for 7.73%, and the chain mediating effect of self-disclosure and social support accounted for 9.87% (**Table 6**).

**Table 4:** Model fitting index.

Model fitting index	Standard or critical value	Results	Judgment of model fitness
GFI	>0.9	0.963	Yes
AGFI	>0.9	0.906	Yes
RMSEA	<0.08	0.072	Yes
NFI	>0.9	0.958	Yes
TLI	>0.9	0.946	Yes
IFI	>0.9	0.974	Yes
CFI	>0.9	0.974	Yes
*CMIN/DF*	1<NC<3	2.512	Yes

**Table 5:** Bootstrap test for coefficient correction of each path.

Path			Estimate	95%*CI*	*P* value
Self- disclosure	<—	Stigma	-0.529	-0.616~-0.436	0.008**
Social support	<—	Self- disclosure	0.757	0.656~0.858	0.006**
Social support	<—	Stigma	-0.147	-0.234~-0.011	0.031*
QOL	<—	Self- disclosure	0.512	0.373~0.584	0.018*
QOL	<—	Stigma	-0.053	-0.176~0.091	0.429
QOL	<—	Social support	0.093	0.057~0.128	0.010**

^*^:p<0.05; ^**^:p<0.01.

**Table 6:** Multiple mediating effect test results.

Path	Estimate	95%*CI*	*P* value	Effect (%)
Total effect	-0.375	-0.499~-0.246	0.005**	—
Direct effect	-0.053	-0.176~0.091	0.429	14.133
Indirect effect	-0.322	-0.421~-0.234	0.006**	85.867
Stigma→Self- disclosure→QOL	-0.271	-0.345~-0.195	0.008**	72.267
Stigma→Social support→QOL	-0.014	-0.028~-0.003	0.013*	3.733
Stigma→Self-disclosure→Social support→QOL	-0.037	-0.057~-0.022	0.008**	9.867

^*^:p<0.05; ^**^:p<0.01.

## 4 Discussion

### 4.1 QOL in patients with breast cancer after operation

This research has shown that the median total score of QOL was 88 points (range 0-144 points), which is above average. Compared with the research of Criscitiello C et al. (23), the QOL in this study is slightly lower (99.0 ± 21.9). Lu Q et al. (24) showed that compared with their American counterparts, Chinese breast cancer survivors reported a lower QOL. However, in recent years, through effective intervention measures, the QOL of breast cancer patients had been greatly improved (7), and the QOL in this research has exceeded the average level, but it is still slightly lower than that of breast cancer patients in other countries (23). It may be that most of the participants in this study have a low education level, medium economic status, and younger age structure, making them more prone to panic about death, unknown, and financial contraction, which would reduce the quality of life (7, 25). The majority of participants undergo mastectomy (62.7%) and have the lowest score in the functional status dimension of FACT-B (**Table 2**). Patients with mastectomy are more likely to suffer from physical and psychological discomfort, and their probability of shoulder and motor function limitation is 6 times higher than that of patients with breast-conserving surgery (26). The early postoperative functional score is also lower than that of patients who chose breast-conserving, and the systemic side effects are more severe (24, 27). Therefore, similar to the studies in China (28, 29), the QOL of the participants in this study is above average, in which the score of functional status is the lowest, but the QOL was still slightly lower than that of foreign breast cancer patients.

### 4.2 Relationship between stigma and QOL in patients with breast cancer after operation

This research explores the relationship between stigma and QOL and its influence path in patients with breast cancer after surgery. Among them, the stigma of patients is above average, which is similar to most studies, indicating that patients with breast cancer after surgery generally experience stigma (8, 9). The results show that stigma is negatively correlated with QOL and its dimensions (**Table 3**). By constructing a structural equation model (**Figure 1**), we further explain the path of stigma affecting QOL: the direct effect of stigma on QOL is not significant, and the way of influence is mainly through indirect effects. Similar to previous studies, there was a negative correlation between stigma and QOL (4, 10), and stigma prevented patients from seeking medical help and adhering to treatment (10, 30). For cancer patients, it is an obstacle to maintaining health-related QOL (31, 32). Hatzenbuehler ML et al. (33) proposed that there were mediating effects regulated by different mechanisms between stigma and QOL. According to different mediating effects, there is a direct effect between stigma and QOL (34), and it can also be completely affected by indirect effects (8). In this study, the effect of stigma on QOL is completely mediated by self-disclosure and social support.

### 4.3 Chain mediating effect of self-disclosure and social support between stigma and QOL in breast cancer patients after operation

In this survey, self-disclosure and social support of patients after breast cancer surgery are negatively correlated with stigma and positively correlated with QOL (**Table 3**). The results of **Figure 1** and **Table 6** show that there are three indirect effects of stigma on QOL: stigma→self-disclosure→QOL; stigma→social support→QOL; stigma→social support→QOL; stigma→self-disclosure→social support→QOL.

#### 4.3.1 Mediating effect of social support between stigma and QOL

The results of this study show that the indirect effect value of stigma → social support →QOL is -0.014, accounting for 3.73% of the total indirect effect. The stigma experience can improve the QOL through the increase of social support. The breast cancer patients who have finished surgery often have social support needs (35), when patients receive more social support, their stigma would be lower (36). Social support can improve the QOL of patients (12, 13), and buffer the pressure by promoting their mental health and physical health (36, 37). At the same time, based on the theory of “stress buffer hypothesis”, social support can be used as a “ direct driver” to improve personal well-being and health, and as a “pre-factor” to improve individuals’ positive coping styles and psychological status, so as to have a positive impact on life and health (4). Therefore, while paying attention to the physical condition of breast cancer patients after surgery, medical staff should give more care to patients, mobilize their families and friends to support them, understand their inner feelings and needs, and give feedback timely.

#### 4.3.2 The chain mediating effect of self-disclosure and social support between stigma and QOL

The results of this study show that the indirect effect value of stigma → self-disclosure →QOL was -0.271, accounting for 84.16% of the total indirect effect, ranking first among the three mediating effect paths. Chinese breast cancer patients believe that coping with disease and misfortune is a private matter, and they are reluctant to disclose their diagnosis, treatment, and disease-related thoughts and feelings to others (38). Women who do not disclose their diagnosis and related concerns are more likely to blame themselves, which may increase the risk of depression that affected emotional well-being and reduce the QOL (39). According to the “social cognitive processing theory”, individual adaptation to cancer can be facilitated by emotional disclosure, which helps to improve psychological adaptation to cancer in the social environment (40, 41). The results show that the indirect effect value of stigma→self-disclosure→social support→QOL was -0.037, accounting for 9.87% of the total indirect effect. Similar to the path of this research, the results of Taniguchi E et al. (15) showed that the characteristics of self-disclosure implied stigma and indirectly promoted psychological well-being through social support, which was a prerequisite for social support. At the same time, self-disclosure enhanced the benefits of social support and was a “booster” of social support. The research of R Rüsch N et al. (42) showed that the better family and friends’ attitude towards patients’ self-disclosure, the better the QOL of patients. Therefore, when medical staff is concerned about the stigma experienced by patients after breast cancer surgery, we should encourage patients to express their emotions and also encourage their families and friends to respond effectively to the expression, express their concern and support for patients, then improve the QOL of patients as much as possible.

To sum up, the QOL of breast cancer patients after surgery still needs to be improved, which can be affected by stigma, self-disclosure, and social support. Stigma can affect QOL through multiple mediating effects of self-disclosure and social support. To improve the QOL of patients, we should encourage patients and their families to express themselves and carry out relevant psychological counseling activities.

### 4.4 Study limitations

There are some limitations to this study. First, this study uses a cross-sectional design, only the independent time point data are collected, and it can not assess patients at different times. A longitudinal study design can be introduced in the later research to explore the trajectory of patients’ QOL at different times. Secondly, this study adopted a convenient sampling method to collect data in five hospitals, and the representativeness of the sample needs to be improved. Although we used convenient sampling, we tried our best to achieve “stratified sampling” by hospital and operation. In the future, we will continue to expand the sample size, increase the hospitals included in the study, adopt a random sampling method, and include more influencing factors for analysis to obtain more accurate conclusions.

## 5 Conclusions

The QOL of patients with breast cancer after surgery is at the upper middle level, which is higher than before, but it can still be improved compared with other countries. Stigma, self-disclosure, social support, and QOL are correlated with each other, and self-disclosure and social support play a multiple chain mediation effects between stigma and QOL. To improve the QOL of patients with breast cancer after surgery, medical staff should not only pay attention to the physical condition of the patients, but also pay attention to the evaluation of their stigma experience, encourage patients to express their emotions, and also encourage their families and friends to respond to the expression and needs of the patients.

## Data availability statement

The original contributions presented in the study are included in the article/supplementary material. Further inquiries can be directed to the corresponding authors.

## Ethics statement

All methods were performed in accordance with the relevant guidelines and regulations or declaration of Helsinki. The study was approved by the ethics committee of Shantou University Medical College (Approval No: SUMC-2021-54). Informed consent was obtained from all individual participants and legal guardian. Written informed consent was obtained from the individual(s) for the publication of any potentially identifiable images or data included in this article.

## Author contributions

All authors contributed to the study conception and design. Material preparation, data collection and analysis were performed by LB, SZ and GF. The first draft of the manuscript was written by LB and all authors commented on previous versions of the manuscript. GF ultimately modified the manuscript. All authors read and approved the final manuscript.
